# A system for accurate and automated injection of hyperpolarized substrate with minimal dead time and scalable volumes over a large range^☆^

**DOI:** 10.1016/j.jmr.2013.10.024

**Published:** 2014-02

**Authors:** Steven Reynolds, Adriana Bucur, Michael Port, Tooba Alizadeh, Samira M. Kazan, Gillian M. Tozer, Martyn N.J. Paley

**Affiliations:** a Academic Unit of Radiology, University of Sheffield, Sheffield S10 2JF, United Kingdom; b MRI Unit, Biomedical Services Unit, University of Sheffield, Sheffield S10 2TN, United Kingdom; c Tumor Microcirculation Group, Department of Oncology, CR-UK/YCR Cancer Research Centre, University of Sheffield, Sheffield S10 2RX, United Kingdom

**Keywords:** Hyperpolarization, *In vivo* automated injection system, Large volume range, Dead space minimization

## Abstract

Over recent years hyperpolarization by dissolution dynamic nuclear polarization has become an established technique for studying metabolism *in vivo* in animal models. Temporal signal plots obtained from the injected metabolite and daughter products, e.g. pyruvate and lactate, can be fitted to compartmental models to estimate kinetic rate constants. Modeling and physiological parameter estimation can be made more robust by consistent and reproducible injections through automation. An injection system previously developed by us was limited in the injectable volume to between 0.6 and 2.4 ml and injection was delayed due to a required syringe filling step. An improved MR-compatible injector system has been developed that measures the pH of injected substrate, uses flow control to reduce dead volume within the injection cannula and can be operated over a larger volume range. The delay time to injection has been minimized by removing the syringe filling step by use of a peristaltic pump. For 100 μl to 10.000 ml, the volume range typically used for mice to rabbits, the average delivered volume was 97.8% of the demand volume. The standard deviation of delivered volumes was 7 μl for 100 μl and 20 μl for 10.000 ml demand volumes (mean S.D. was 9 ul in this range). In three repeat injections through a fixed 0.96 mm O.D. tube the coefficient of variation for the area under the curve was 2%. For *in vivo* injections of hyperpolarized pyruvate in tumor-bearing rats, signal was first detected in the input femoral vein cannula at 3–4 s post-injection trigger signal and at 9–12 s in tumor tissue. The pH of the injected pyruvate was 7.1 ± 0.3 (mean ± S.D., *n* = 10). For small injection volumes, e.g. less than 100 μl, the internal diameter of the tubing contained within the peristaltic pump could be reduced to improve accuracy. Larger injection volumes are limited only by the size of the receiving vessel connected to the pump.

## Highlights

• MR compatible injection system that can be operated in high magnetic fringe field.
• Automated rapid, accurate and reproducible multipart injection of hyperpolarized substrate.
• Flow direction control minimizes dead volume in cannula to increase *in vivo* signal.
• Sample mixer provides homogeneity of injected pyruvate, pH 7.1 ± 0.3, mean ± S.D.
• Between 100 μl to 10.000 ml delivered volume was 97.8% of demand.

## Graphical abstract

**Figure:**
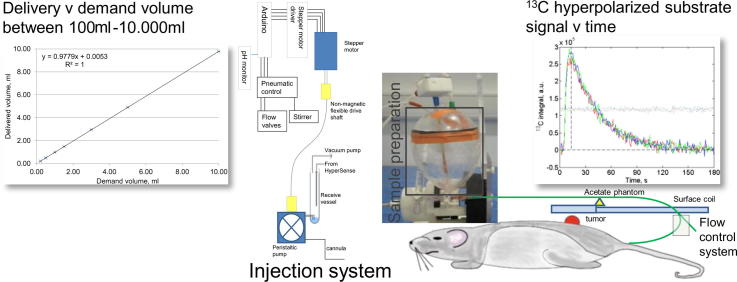

## 1 Introduction

Dissolution dynamic nuclear polarization (dDNP) [1] has afforded a step change in the available MR signal for nuclei such as ^13^C. It has most extensively been applied to hyperpolarized MR imaging and spectroscopy used for the assessment of *in vivo* metabolism, with considerable interest in elucidating the glycolytic pathway, e.g. intravenously injected hyperpolarized ^13^C-pyruvate conversion to lactate. In contrast to conventional (thermally polarized) MR, the hyperpolarized signal is transitory due to *T*_1_ relaxation. This means that the dDNP experiment must be conducted as rapidly as possible, within a few multiples of the *T*_1_ relaxation time, before the signal decay becomes too significant. The hyperpolarized signals are acquired rapidly to provide spectroscopic information on the conversion of the injected substrate to its metabolites within the tissue of interest and has been applied to the imaging of tumors [2] and their response to drug treatment [3]. Further development of the methodology has allowed the temporal signal plots obtained from tissue to be fitted to compartmental models to estimate kinetic rate constants [4]. We have shown previously that a reproducible injection/withdrawal system can be used to provide a consistent arterial input function for compartmental modelling and extraction of physiological parameters [5]. A rapid and reproducible injection regime is also highly desirable for comparative hyperpolarization studies, where a precisely delivered dose to each subject is of prime importance.

A previously developed automated injection system [6] provided reproducible injection volumes, rates and timing for animal studies [5]. However, because of its syringe-based design, it was limited in the range of volumes it could deliver: 0.6–2.4 ml – a volume range typically used for the injection of rats. Also, the injection was delayed by a few seconds because of the syringe filling stage required by this system.

As the hyperpolarized signal lifetime is governed by *T*_1_ relaxation, reducing the delay between dissolution and injection can improve the magnitude of the signal, particularly for short *T*_1_ molecules. Moreover, extending the working range of the injectable volume would allow the application of the injection system to a wider range of species. Other design features for the injection system should ensure homogeneous composition of the final hyperpolarized substrate, coupled with flow control to minimize the dead volume of the injection received by the animal, monitoring of pH and ease of use. Here we show an improved MR compatible automated injector system that fulfils these requirements.

## 2 Materials and methods

### 2.1 Injector design

**Fig. 1:**
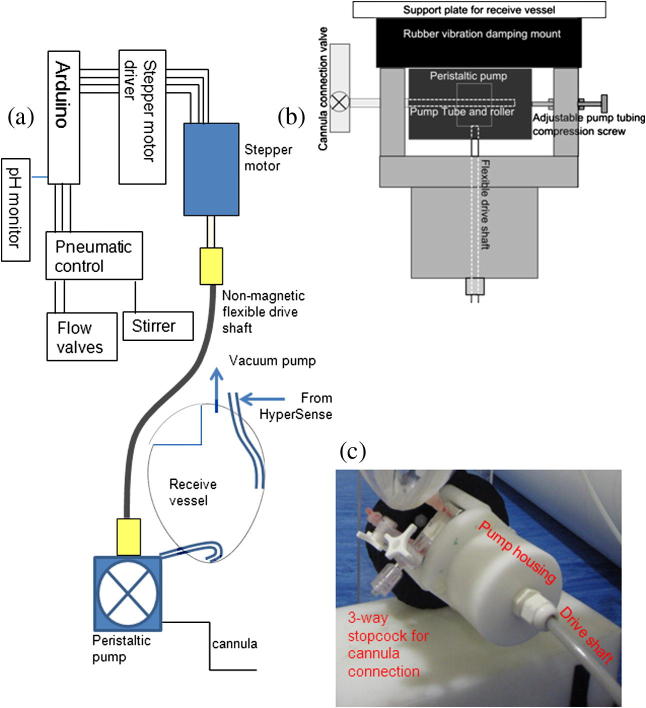
(a) Overview of injector system design showing the control system with the stepper motor, flexible drive shaft and pump and the receive vessel, (b) pump chassis, drive shaft and 3-way stopcock, and (c) image of injector system.

The injector consists of a peristaltic pump directly driven through a flexible drive shaft by a stepper motor. A high torque bipolar stepper motor (57BYG621, Wantai Motor, Changzhou, China) was mounted on to a housing fixed to the magnet room filter plate outside the 5 G line of the magnet, see Fig. 1. The non-magnetic flexible drive shaft was constructed of a 4 mm phosphor-bronze shaft, 2.5 m in length (SS White Technologies Ltd., Milton Keynes, UK), inserted into a 6 mm O.D. nylon tube. The drive shaft was interfaced with a plastic peristaltic pump (150 series, Williamson Pumps Ltd., Poynings, UK) mounted on its own plastic machined polyoxymethylene (POM) chassis. The pump and chassis were bolted to a rubber plate to minimize vibration from the stepper motor and a POM/Perspex support stand was used to form the complete injection system. An adjustable screw was fitted to the rear of the peristaltic pump to vary the degree of compression exerted by the pump housing on the tubing contained within the peristaltic pump. This was done in order to reduce the torque requirement for the drive shaft and stepper motor. The injection system incorporates a receiving vessel, attached to the inlet port of the pump, for collection and neutralization of the substrate to be injected, when this is required. The output of the pump was connected to a 3-way Luer lock stopcock (Becton Dickson) to permit easy connection to an intravenous cannula and, after switching the flow direction, for flushing pipework. Control of the stepper motor and injection system was realized by an Arduino microcontroller, as described below.

### 2.2 Receiving vessel design

**Fig. 2:**
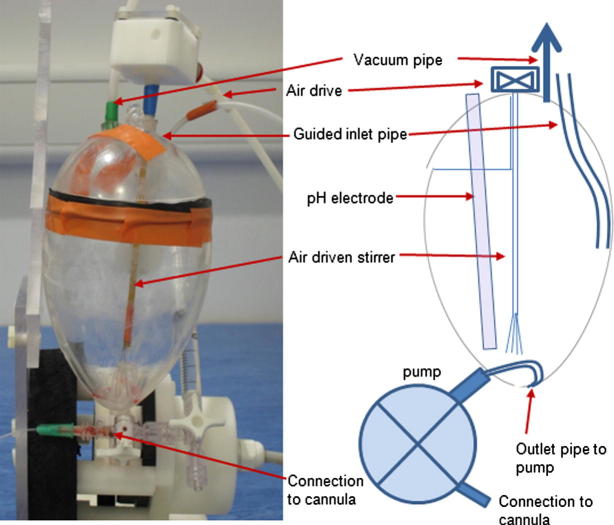
Schematic and image of hyperpolarized fluid receive vessel (not to scale). Shown are the receive vessel with pipe guide (into which the DNP polarizer dissolution pipe was inserted), air driven stirrer, pH electrode and vacuum pipe. Connection to an animal cannula is made via a Luer 3-way stopcock fitted to the outlet of the pump.

Homogeneity and pH of the injected substrate is important for *in vivo* applications. For manual injection of substrate, the operator can agitate the liquid to improve its homogeneity. This is a particular requirement for pyruvic acid which, prior to injection, must be converted to its salt by reacting with a pre-determined aliquot of sodium hydroxide. For an automated system, the design of the device must ensure that this reaction proceeds to completion prior to injection. A custom receive vessel (RV) was designed to ensure smooth flow of liquid into the vessel in order to minimize acid or base splashing on the walls. The RV was constructed from a 120 mm polycarbonate egg shape (Polycraft supplies, Cardiff, UK) machined to permit inlet of services, see Fig. 2. After dissolution, hyperpolarized substrate flows into the RV from the DNP polarizer through a 3 mm O.D. fluorinated ethylene propylene (FEP) pipe that passes into a 6 mm I.D. Tygon guide pipe (Cole-Parmer, London, UK) glued inside the RV vessel wall. The guide pipe allowed consistent positioning of the dissolution pipe half way up the vessel wall. At the end of the FEP pipe was a nozzle to guide the liquid down the RV wall. Hyperpolarized substrate was withdrawn from the RV into the pump via a side port fitted into the lower section of the RV.

In this implementation, a predetermined aliquot of 2.0 M sodium hydroxide was added to the RV prior to ingress of the pyruvic acid. To ensure thorough mixing of pyruvic acid with the sodium hydroxide, an air driven stirrer was inserted into the RV, see Fig. 2. The stirrer was constructed with a POM paddle wheel on a 2 mm diameter fiber glass spindle, 14 cm in length. At the other end of the spindle there were 4 cm horse hair brush fibers which were submerged in the liquid to rapidly stir and homogenize the mixture. The paddle wheel was located inside a housing and driven by an air supply automatically gated on using a pneumatic control valve and the Arduino microcontroller. Also, inside the RV there were placed a few (5–7) 2 mm diameter glass beads that helped to damp liquid motion when pyruvic acid first entered.

The pH of the injected substrate was automatically measured during the dissolution procedure using a pH electrode (ASP200-2-1M-BNC, Active robots Ltd., Radstock, UK) placed in the RV. The pH electrode was connected to a custom built amplifier that had a variable output voltage in response to changes in pH, see Supplementary information. The amplifier was connected to an analogue to digital converter input on the microcontroller. By titrating 45 mg pyruvic acid against 2.0 M sodium hydroxide over a pH range of 1.8-–13.0, voltage versus pH was plotted and used to generate a linear calibration equation. The pH electrode could also be calibrated from within the Arduino software by measuring the electrode voltage in 3 different buffer solutions (pH 4, pH 7, pH 10) and calculating a linear equation for pH versus voltage.

Also connected into the RV was a 6 mm O.D. pipe connected to a vacuum pump (GAST GF3, Gast Manufacturing Inc., MI) to reduce back pressure during transfer of hyperpolarized solution. The vacuum pump was gated on/off by the HyperSense DNP polarizer.

### 2.3 Flow diverter system design

Injection volumes for each species are limited to ensure that the circulation of the animals is not overloaded. The physical constraints of an MRI scanner require a long length of cannula line for i.v. injections, resulting in a significant dead volume that contributes to the injection volume. This is problematic where hyperpolarized signal is limited. If, for example, saline occupies the dead volume of the cannula then, during injection, its volume must be considered part of the dose and yet it does not contribute to the measured hyperpolarized signal. To increase the percentage the hyperpolarized compound contributes to the injected volume, the dead volume was reduced by splitting the cannula into two pathways without introducing additional dead volume; one pathway was then used as a waste stream for clearing the dead space volume whilst the other was used for drug administration into the animal. Flow direction was computer-controlled by valves.

**Fig. 3:**
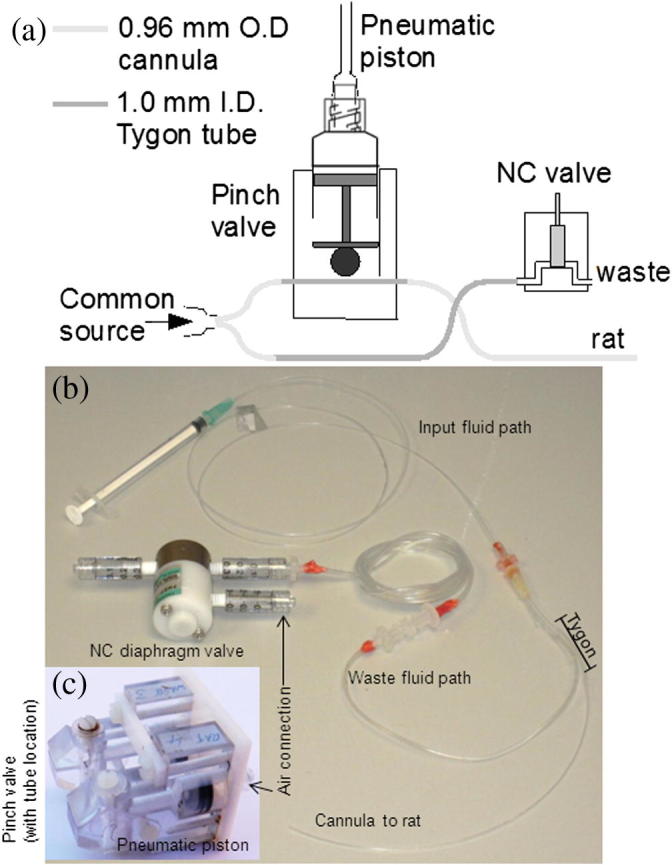
Flow diverter configuration showing: (a) schematic of flow diverter cannula, pinch valve for controlling flow to the rat and normally closed (NC) diaphragm valve, (b) complete fluid path for flow diversion system, and (c) 2 pinch valves.

A fluid diverter cannula was constructed using two types of tubing: 0.96 mm O.D. polyethylene tube (Portex, Smiths Medical, St. Paul, MN), hereby referred to as ‘small tube’ and 1.0 mm I.D. Tygon tube (Cole-Parmer, London, UK), hereby referred to as ‘large tube’. Tygon tubing was used as its mechanical properties permit multiple compressions without permanent damage. A 19 gauge Luer hub was drilled to enlarge its inner diameter to 2 mm, see Fig. 3, into which the ends of three 30 mm lengths of small tubing were inserted to ensure the hole was almost completely occupied; one was used for the waste pathway, one for the animal pathway which was inserted into a rat vein, whilst the third one was unused and blanked off. The tubes were then sealed to the Luer hub with glue. Onto the waste pathway ∼200 mm of large tubing was glued and terminated with a Luer 3-way stopcock to prevent animal bleeding during surgery. The animal pathway was continued with a short 30 mm piece of large tubing followed by a 180 mm length of small tube that would be used to cannulate the animal. To allow access from outside the magnet, an ∼800 mm length of small tubing was attached upstream of the two-way cannula with a 21 gauge luer hub glued at one end and with the other end potted into a male–male Luer adapter (Cole-Parmer) using dental cement, see Fig. 3.

Fluid flow pathway was guided using a custom-made pinch valve and a normally closed diaphragm valve (PMDP-2R-M6G, Takasago, Nagoya, Japan), both actuated by pneumatic pressure and controlled by an Arduino microcontroller. A custom made pinch valve chassis was machined from polycarbonate (PC) with a 10 mm syringe/plunger that actuated a PC cylinder that acted on the Tygon tube, see Fig. 3. When air pressure was applied either one or both valves could be opened or closed. Air pressure was supplied by a custom made pneumatic control box with independent control valves that could be turned on by a 5 V input provided by the Arduino microcontroller.

Once the diverter cannula had been surgically inserted into the animal and the animal transferred to the magnet, the animal pathway line was inserted into the pinch valve (at the Tygon position) and held in place by a plastic gate. The waste line of the fluid path was connected to the diaphragm valve and the 3-way stopcock opened to provide a continuous fluid outlet path to waste outside of the magnet.

On injection, the Arduino microcontroller was programed to open the waste line valve and close the rat line valve and pump. Typically 0.6–0.8 ml of liquid went to waste. The fluid path was then switched to the rat by opening the rat line valve and closing the waste line and the desired injection volume was delivered to the rat. During idle mode, the rat side valve was left open to prevent damage to the Tygon tube.

### 2.4 Injector control system

The injector pump system was controlled through a computer serial link to an Arduino Uno R3 microcontroller (Arduino.cc). The microcontroller controlled the stepper motor via custom made stepper motor driver electronics. The flow diverter and air stirrer were operated via a custom made pneumatic control box which provided air pressure (pre-set at 40–60 PSI) in response to 5 V input signals. Trigger of the injection sequence was started in response to a 5 V signal (greater than 10 ms duration to eliminate false triggering) from either the HyperSense polarizer or scanner console to the microcontroller. Once the injection system had been placed in trigger standby, the pH in the RV was reported every 30 s for up to 2 min 10 s (a duration chosen to be ∼20 s shorter than the polarizer’s dissolution solvent heating preparation time). After 2 min 10 s the pH was reported every 1 s in order to record the neutralization of pyruvic acid by sodium hydroxide, see below. On receiving a trigger signal a final pH was reported. A software option was available to gate whether the injection proceeded if the pH was within a predefined range, e.g. pH 7 ± 1. The reported pH was estimated to be accurate to ±0.1.

Other devices requiring external device control could be connected to the available external digital output pins of the Arduino board.

User-programed software functions controlled the activity of the pump, permitting multistep flow rates, volumes (absolute or calculated volume as a function of animal weight for a given dose) and flow direction. Volume calculations were made by the software using a calibration volume based on a single revolution by the stepper motor. Using a software function, the calibration volume could be determined based on the mass of water delivered after ten revolutions of the stepper motor or by adaptive volume calibration, in which the calibration volume was internally adjusted based on the measured volumes and compared against the requested volume by the software. Additional features included a cleaning routine using flow control to flush the pump and cannula whilst still connected to the animal.

### 2.5 Volume delivery calibration and test

The pump was first calibrated by three measurements of the mass of distilled water delivered through 1100 mm of 0.96 mm O.D., 0.58 mm I.D. tube into a glass vial after ten revolutions of the pump at 80 rpm. The average water mass divided by ten was then entered into the software as a volume per revolution. All subsequent volumes were calculated by the software based on this calibration. The accuracy and scalability of the injection system delivered volumes were measured against programed volumes in the range 0.100–10.000 ml for an arbitrarily chosen constant flow rate of 7.0 ml/min. The delivered volume for a given demanded volume was similarly measured at least three times (range 3–5) by mass of distilled water.

### 2.6 Hyperpolarized ^13^C_1_ pyruvic acid experiments

The delivery of hyperpolarized substrate was tested firstly *in vitro* and subsequently *in vivo*. In both types of experiments ^13^C_1_ pyruvic acid (PA) (Sigma Aldrich, Gillingham Ltd., UK) was mixed with 15 mM OX63 trityl radical (Oxford Instruments, Abingdon, UK) and 1.5 mM DOTAREM (Guerbet, Roissy, France). 45 mg (12.7 mg for *in vitro* tests) of PA was hyperpolarized using a HyperSense DNP polarizer, operating between 1.2 and 1.4 K, using a microwave frequency 94.150 GHz and 30 mW for approximately 1 h. The hyperpolarized frozen sample was transferred to the receive vessel using 3.4 ml superheated buffer solution containing 40 mM HEPES buffer solution, 0.269 mM disodium EDTA and 50 mM NaCl (all obtained from Sigma Aldrich). The receive vessel contained a predetermined aliquot of 2.0 M sodium hydroxide solution (Sigma Aldrich) required to neutralize the PA and 2.0 ml HEPES/EDTA buffer solution to ensure that the receive vessel outlet pipe was submerged. Final concentration of PA was ∼100 mM. The transfer time of the hyperpolarized solution from the polarizer was 6 s, after which a trigger signal was sent to the Bruker 7T small animal scanner (310 mm horizontal bore, with 400 mT/m, 120 mm I.D. gradient insert, Bruker Biospin MRI GmbH, Ettlingen, Germany) to commence acquisition and start the injection procedure.

### 2.7 *In vitro* experiments

Two separate experiments were performed to test the delivery and reproducibility of the injection system. First, for volume delivery, an injection was performed using hyperpolarized ^13^C pyruvate into a plastic vial mounted on a 20 mm ^13^C/^1^H surface coil (Bruker) placed at the center of the magnet. Second, a 0.96 mm O.D. cannula tube was attached to the injector and positioned so that it ran in a straight horizontal direction across the face of the surface coil, parallel to the *z*-axis at a distance of 5 mm from the coil surface. This configuration was undisturbed for three consecutive injections. In both experiments the injection was programed to deliver 1.50 ml of pyruvate at 6.92 ml/min, simultaneously starting with the MR acquisition sequence. A 6.7 M acetate phantom was attached on the other side of the coil to provide a reference signal. The ^13^C signal was localized using a 20°, 0.5 ms Gaussian pulse and 10 mm slice selection. 180 consecutive spectra (sw = 50 ppm, 256 points) were acquired with a TR = 1 s; 180 s total duration. Integrals were measured from spectra using custom Matlab software (MathsWorks Inc., Natick, MA).

### 2.8 *In vivo* experiments

Animal experiments were conducted in accordance with the United Kingdom Animals (Scientific Procedures) Act 1986, with local ethical approval and following published guidelines for the use of animals in cancer research [7].

**Fig. 4:**
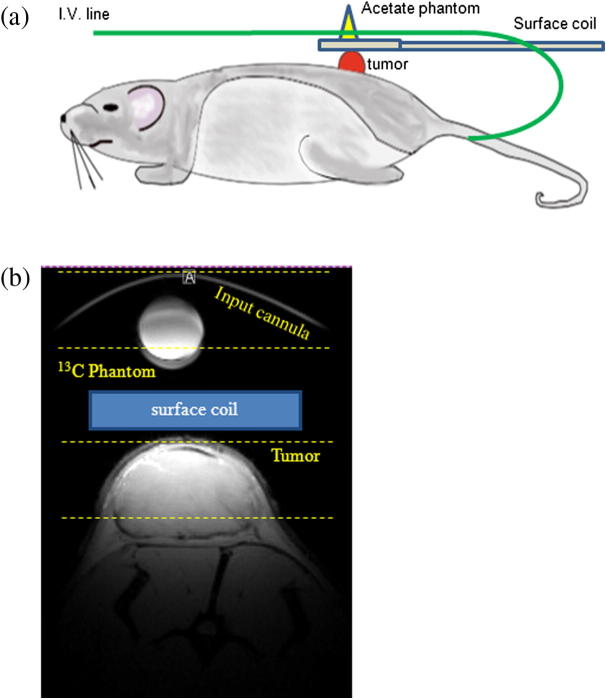
(a) Sketch of rat showing location of surface coil and routing of i.v. cannula, (b) axial FLASH image of the tumor, reference phantom and femoral vein cannula. A cartoon representation (blue box) of the surface coil location and guide lines for 2 slices are also shown. (For interpretation of the references to color in this figure legend, the reader is referred to the web version of this article.)

BDIX rats, with subcutaneously implanted P22 tumors, were anaesthetized with 1.5–2% isoflurane at 2 L/min via a nose cone and the tail vein was cannulated for ^13^C_1_-pyruvate (PA) delivery. The rat was placed in a Bruker 7T MRI system with its temperature maintained by an electric heating pad and rectal temperature probe. Respiration rate was also monitored. A 20 mm ^13^C/^1^H surface coil was placed 1–2 mm above the tumor, with the I.V. tail vein diverter cannula routed over the top of the surface coil to provide an *in vitro* reference signal, see Fig. 4a. ^13^C signals were localized in the tumor by 8 mm coronal slice selection with a 20° 0.5 ms Gaussian pulse. All other acquisition parameters were the same as the *in vitro* experiment. 5 ml/kg of hyperpolarized PA at ∼100 mM was administered over 13 s using the injection system and the flow diverter. From the resulting spectra ^13^C pyruvate peak integrals versus time response curves were processed using Matlab.

To locate the slice positions for the hyperpolarized PA experiments, structural images of the tumor were acquired with the 20 mm ^13^C/^1^H surface coil using a FLASH sequence (FOV 60 × 60 mm, 256 × 256 matrix, 13 slices, 1 mm thickness, TR/TE 164.71/6 ms). A representative image is shown in Fig. 4b.

## 3 Results

### 3.1 Volume delivery correlation with demand volume

**Fig. 5:**
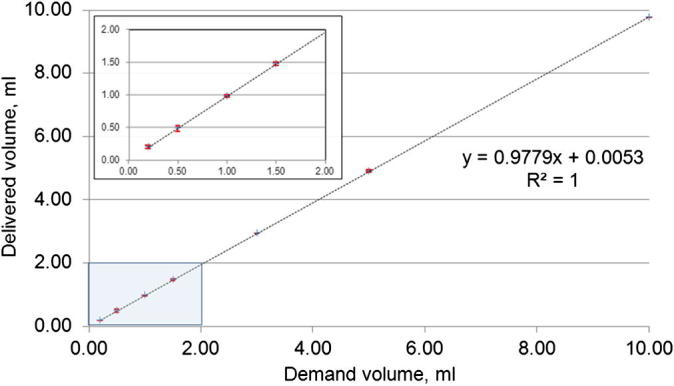
Demand volume versus average delivered volume for at least 3 measurements by mass of water. Error bars represent the standard deviation in delivered volume. Inset shows zoomed region of main figure in the volume range 0.00–2.00 ml.

The reproducibility of the injection volume was tested by measuring the mass of water delivered. Delivery times for 100 μl to 10.00 ml volumes at a constant flow rate of 7 ml/min ranged from 0.9 to 86 s. Delivered volumes versus the demand volumes were plotted in Fig. 5. An excellent linear correlation was found between the demand and delivered volumes, from 100 μl to 10.00 ml with *r*^2^ = 1. Across the entire measured range, the mean delivered volume was found to be 97.8% of the demanded volume. The standard deviation of delivered volumes was 7 μl for 100 μl and 20 μl for 10.000 ml demand volumes (mean S.D. was 9 μl in this range).

### 3.2 Hyperpolarized experiments

#### 3.2.1 *In vitro* test

**Fig. 6:**
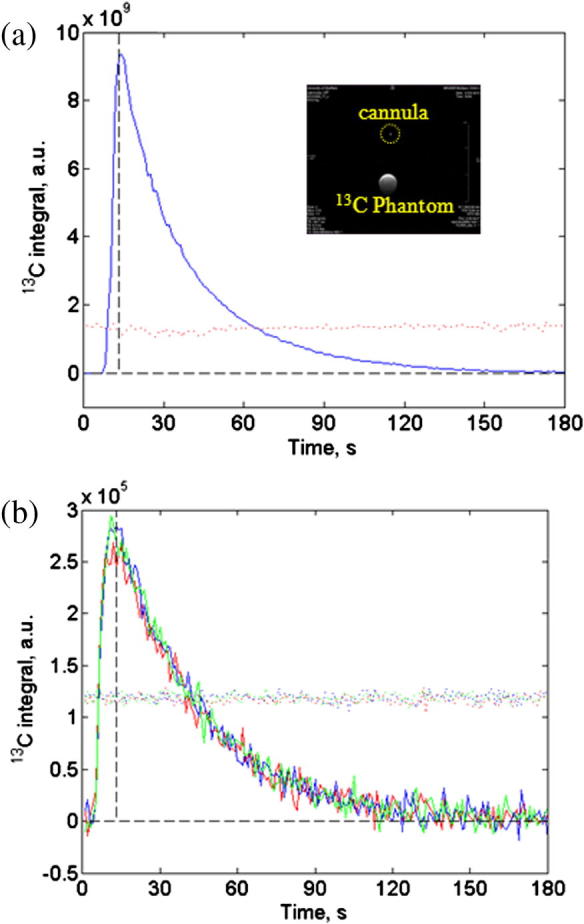
*In vitro* delivery profile for injection of 1.50 ml of hyperpolarized pyruvate over 13 s as measured by the integral of the ^13^C PA signal. Temporal data acquisition commences simultaneously with injector start. 6.7 M ^13^C sodium acetate used as signal reference; (a) single injection into vial mounted on a 20 mm ^13^C/^1^H surface coil, (b) 3 repeat injections through a fixed horizontal tube located 5 mm from ^13^C surface coil surface (color matched solid lines pyruvate signal, dotted colored lines acetate reference. Vertical dashed line at 13 s). (For interpretation of the references to color in this figure legend, the reader is referred to the web version of this article.)

By acquiring the ^13^C MR signal, the delivery profile of 1.5 ml of hyperpolarized pyruvate at 6.92 ml/min (13 s pumping time) was measured as it was injected into a plastic vial, see Fig. 6a. ^13^C spectra acquisition started simultaneously with the pump after a trigger signal from the HyperSense. The first detected signal in the vial appeared at 6 s after the injection started, with the maximum signal observed at 13 s – coinciding with the end of the injection time point. For three repeat injections through a fixed tube, the overlaid absolute integral plots closely matched each other, see Fig. 6b. From measurements of the area of each curve the coefficient of variation was 2%.

### 3.3 *In vivo* DNP/MR experiments

**Fig. 7:**
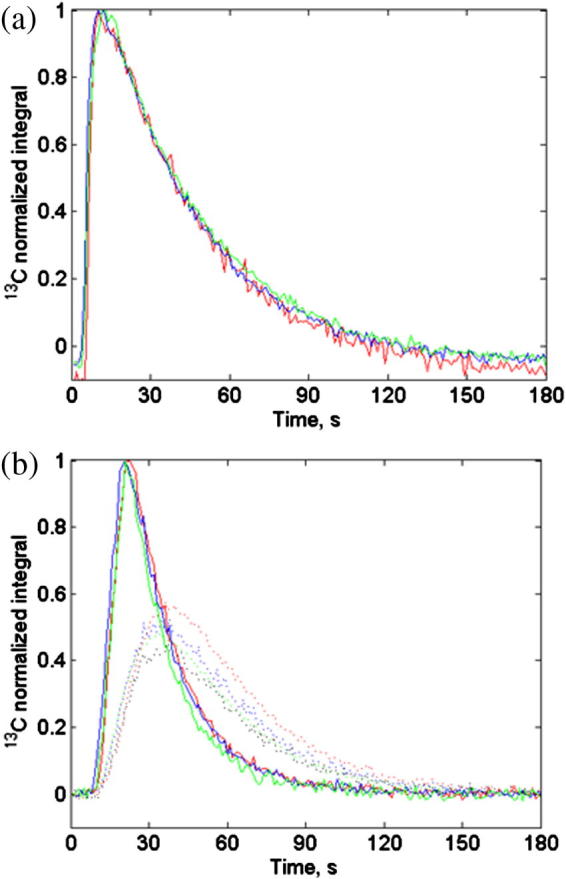
Normalized integral for hyperpolarized ^13^C pyruvate versus time for 5 ml/kg injection over 13 s in three rats, (a) ^13^C signal from tail vein cannula and (b) slice localized ^13^C signal from tumor (dotted lines – lactate signal). In both figures the traces are color matched for individual animals. (For interpretation of the references to color in this figure legend, the reader is referred to the web version of this article.)

After the pump system had been tested *in vitro* it was then employed for *in vivo* injections over 13 s into P22 sarcoma bearing BDIX rats using the flow diverter system. With the surface coil positioned over the tumor, ^13^C spectra were simultaneously acquired both from the tumor and from the tail vein cannula located above the surface coil. Fig. 7a shows that the ^13^C signal from the tail vein cannula signal first appeared at 3–4 s after the trigger signal started the signal acquisition and injection sequence, reflecting the time of flight of hyperpolarized substrate through the pump and cannula to the rf coil. The tumor pyruvate signal first appeared at 9–12 s after the trigger signal and reached maximum at 21–23 s. There was approximately 13 s between appearance and maximum signal in the observed tumor, closely matching the period of injection. The pH (measured post-injection using IQ150 pH meter, Hach Company, Loveland, CO) of the injected pyruvate was 7.1 ± 0.3 (mean ± S.D.) for 10 animals.

## 4 Discussion

The design of the injection system permitted reproducible administration of hyperpolarized substrate with minimal human intervention. The plastic/non-ferrous construction of the injector allows it to be positioned next to the bore of the magnet (tested at fringe magnetic field strengths of ∼1 T). In this implementation the drive shaft length was chosen so that the drive motor was outside the 5 G line. The drive shaft could be shortened or lengthened in accordance with magnet room layout, although care must be taken over choice of the diameter of longer drive shafts to prevent excessive twisting. An excellent correlation between demanded and delivered volume was found for the tested volumes: from 0.100 to 10.00 ml, a volume range that is typically used for mice and larger animals such as rabbits. Delivered volume was also highly reproducible over this large volume range. Should smaller volumes, e.g. less than 100 μl, be required then the internal diameter of the tubing contained within the peristaltic pump could be reduced to improve accuracy.

Over the past few years a number of different solutions have been designed to address reproducibility in delivery of hyperpolarized substrate. A system by Bowen and Hilty [8] was designed for *in vitro* use to rapidly (1200 ms) inject hyperpolarized dissolute into a high resolution NMR spectrometer. Specifically their system used high pressure, >40 bar, to ensure that an aqueous solution reliably filled a 5 mm NMR tube without air bubbles – a common issue due to the high viscosity and surface tension of water. Due to its high operating pressure their design would not be readily applicable to *in vivo* use without stepping down the pressure.

A computer controlled *in vivo* injector was described by Comment et al [9], further improved in [10], that addressed the issue of bubble formation by allowing the chase gas (used to assist transfer of the sample from the polarizer to the injector) to exit through vents. A hydraulically driven plunger then sealed the vent holes as the sample was injected into the animal. An in-line optical sensor halted the injection if a bubble was detected within the injection cannula. The presence of a vent hole affects the accuracy of such a system, as there would be some variability in the amount of liquid injected into the animal as these vents were sealed. Hydraulic based systems also have some inaccuracy due to friction in actuating the hydraulic cylinder(s). In our described system, the possibility of injecting an air bubble was minimized by having a continuous fluid path from the cannula to the RV. The outlet pipe of the RV to the pump was also always submerged. The ingress of hyperpolarized substrate passed down the side of the RV wall to smoothly fill the RV and a vacuum pump removed excess gas. In practice, no bubbles were found to have formed within the RV and so this was not regarded as a safety issue. However, an optical bubble detection system, as described [9], could be added and operated with the flow diversion system described here to prevent accidental injection of air into the animal. The design of the RV would permit other quality control systems, similar to those used in a clinical DNP polarizer [11], e.g. volume, temperature, free radical concentration sensors, to be added. Although not included on the current injector, an electrical or chemical heating system would prevent administration of relatively cold substrate to the animal. This would be due to the reduced temperature of the hyperpolarized substrate as it passes through the cannula to the animal while in the room temperature magnet bore (14 °C). Injection of cool substrate has been observed by us to cause an approximate 0.5 °C temperature drop in the animal, as measured by a rectal temperature probe (data not shown), although it is not yet known whether this influences substrate delivery to subcutaneous tumors or pyruvate kinetics. Inclusion of a pH electrode allows online monitoring of the hyperpolarized substrate during the dissolution process to provide additional animal safety. A glass pH electrode can take up to 30 s to attain a stable value, although an approximate value can be measured within a few seconds. Because of this, using the built in pH monitor introduced a few seconds delay to the injection and so was not always used.

The delay between dissolution and injection has been minimized by using a peristaltic pump to remove the syringe filling delay required for a previous automated injector design [6]. The reduction of dead time from dissolution to start of the injection is a key factor in the ^13^C MR studies of hyperpolarized substrates. Saving 1–5 s, depending on the required syringe filling volume, can be an important improvement in terms of experimental sensitivity. Moreover, automation of a combined polarizer and injection system, as seen in the *in vitro* results, can produce a very high degree of consistency in the level of the hyperpolarized signal by fixing the timing and dose of the substrate. The *T*_1_ of hyperpolarized pyruvate has been shown to be highly dependent on magnetic field strength [12] thus affecting the observed level of signal. The injection system can be reproducibly positioned next to the magnet such that the sample experiences a well-defined magnetic field path during transfer from the polarizer. In principle the observed *in vivo* signal can be corrected for timing differences using *T*_1_. However, *in vivo* values of *T*_1_ have been published in the range 18–31 s [13,14], making this method potentially inaccurate. Provided that the injection cannula was consistently positioned with respect to the surface coil, the level of hyperpolarized signal between injections could be measured to assess reproducibility. Combining this measurement with a reference phantom signal would allow the polarization to be calculated. Measuring the hyperpolarized signal in the cannula would cause a reduction of the signal acquired from the animal. However, this measurement could be delayed until after the substrate had been fully administered.

When the injection system was used *in vivo*, the ^13^C MR signal could be first detected within the tissue of interest 8–12 s after transfer of hyperpolarized pyruvate from the polarizer, minimizing hyperpolarization loss and therefore improving the available signal. Variations in the appearance time of the ^13^C signal in the tumor are most likely caused by differing blood circulation times and tumor vascularity between animals.

By using a fully programmable microcontroller, the operation of the injector can be customized to the user’s needs. The consistency of injection flow rate and timing by computer-control ensures a reproducible dose to the animal, allowing hyperpolarized signal to be more readily compared across experiments. While keeping important attributes of the previously developed injector, e.g. reproducible injection volume and flow rate through automation, the new system provides an improvement in the speed, reproducibility, accuracy and scalability of the volume that can be delivered.
