# Supplementary material for: A system for accurate and automated injection of hyperpolarized substrate with minimal dead time and scalable volumes over a large range
Source: J Magn Reson. 2014 Feb;239(100):1–8. doi: 10.1016/j.jmr.2013.10.024 (PMC3969585; doi:10.1016/j.jmr.2013.10.024)
Supplement: Supplementary data 1 [file mmc1.docx]

**Supplementary information**

The circuit design for pH monitoring was adapted from on a previously published project (<http://www.sparkyswidgets.com/portfolio-item/ph-probe-interface/>). Additional components were added to the circuit board to provide ±5 V from a 9 V battery.
